# Intraocular Lens Power Calculation Formulas in Children—A Systematic Review

**DOI:** 10.3390/jcm13154400

**Published:** 2024-07-27

**Authors:** Wiktor Stopyra, Andrzej Grzybowski

**Affiliations:** 1 MW-Med Eye Centre, 31-416 Krakow, Poland; 2 Department of Medicine, University of Applied Sciences, 34-400 Nowy Targ, Poland; 3 Institute for Research in Ophthalmology, Foundation for Ophthalmology Development, 61-553 Poznan, Poland; 4 Department of Ophthalmology, University of Warmia and Mazury, 10-720 Olsztyn, Poland

**Keywords:** intraocular lens power calculation formulas, pediatric cataract, phacoemulsification, Barrett Universal II

## Abstract

**Objectives:** The selection of an appropriate formula for intraocular lens power calculation is crucial in phacoemulsification, particularly in pediatric patients. The most commonly used formulas are described and their accuracy evaluated in this study. **Methods:** This review includes papers evaluating the accuracy of intraocular lens power calculation formulas for children’s eyes published from 2019–2024. The articles were identified by a literature search of medical and other databases (Pubmed/MEDLINE, Crossref, Google Scholar) using the combination of the following key words: “IOL power calculation formula”, “pediatric cataract”, “congenital cataract”, “pediatric intraocular lens implantation”, “lens power estimation”, “IOL power selection”, “phacoemulsification”, “Hoffer Q”, “Holladay 1”, “SRK/T”, “Barrett Universal II”, “Hill-RBF”, and “Kane”. A total of 14 of the most recent peer-reviewed papers in English with the maximum sample sizes and the greatest number of compared formulas were considered. **Results:** The outcomes of mean absolute error and percentage of predictions within ±0.5 D and ±1.0 D were used to assess the accuracy of the formulas. In terms of MAE, Hoffer Q yielded the best result most often, just ahead of SRK/T and Barrett Universal II, which, together with Holladay 1, most often yielded the second-best outcomes. Considering patients with PE within ±1.0 D, Barrett Universal II most often gave the best results and Holladay 1 most often gave the second-best. **Conclusions:** Barrett Universal II seems to be the most accurate formula for intraocular lens calculation for children’s eyes. Very good postoperative outcomes can also be achieved using the Holladay 1 formula. However, there is still no agreement in terms of formula choice.

## 1. Introduction

The global prevalence of pediatric cataract ranges from 1 to 15 per 10,000 children. Moreover, pediatric cataract contributes to 5% to 20% of cases of childhood blindness and severe visual impairment globally, with an incidence rate between 1.8 and 3.6 per 10,000 children annually [1].

Patients’ expectations for acute vision after cataract surgery continue to grow; therefore, the accurate calculation of intraocular lens (IOL) power is a fundamental issue [2]. However, according to European Registry of Quality Outcomes, the percentage of eyes with a prediction error (PE) within ±0.5 D after cataract surgery is only 73.7% [3]. The precision of the artificial IOL power is contingent on the accuracy of the preoperative biometric data, e.g., axial length (AL), keratometry (K), and anterior chamber depth (ACD), but most of all, it relies on the evaluation of effective lens position (ELP) based on the choice of the proper formula [4].

In children, however, the prediction accuracy is significantly lower than it is in adults, with only 66.0% of eyes achieving ±1.0 D SE of target refraction [5]. The difficulty in obtaining a precise IOL power calculation in children results from several causes. First, compared to adults, pediatric eyes have a shorter AL, steeper corneal curvature, higher K value, and shallower ACD [6]. Second, inaccuracy of biometric measurements can be caused by the poor cooperation and fixation of the child [7]. Third, the ELP may be affected by the need for anterior vitrectomy in young children [8]. Fourth, the IOL power calculation formulas applied in children were derived from data from adult eyes, which may be unsuitable for application in pediatric eyes [9].

Moreover, postoperative target refraction in children remains controversial due to the growth of the pediatric eye [7]. To achieve a final refraction close to emmetropia at the end of eye growth and to prevent significant myopic shift, the calculated IOL power must be adjusted intraoperatively for the chosen IOL [10]. Different approaches have been used to address this issue over the years. Initially, eye surgeons aimed for emmetropia at the time of surgery to facilitate treatment of amblyopia. However, when eye growth was complete, the children had experienced large myopic shifts and frequently needed IOL exchange [11]. Hence, in 1997, the first guidelines were proposed for the calculation of IOL power with correction. It was found that initial hypermetropia was not amblyogenic, and it was recommended that surgeons aim for 80% of the IOL power needed for emmetropia for patients under 2 years of age and that they aim for 90% for patients between 2 and 8 years old [12]. One year later, Enyedi suggested more precise guidelines with different target refractions according to the age of the patient at implantation, as follows: +6.0 D at 1 year old, +5.0 D at 2 years old, etc., until +1.0 D at 6 years old; for patients older than 8 years, the adjustment was −1.0 D to −2.0 D [13]. This rule, with minor amendments by various practitioners, is still used today [14,15]. However, sometimes, the outcomes are still not satisfactory [16].

At least several dozen IOL power calculation formulas have been developed to date. They utilize different variables and operate on various principles. However, only some of them are used for children [8,17,18,19,20,21]. The aim of this paper is to describe and to compare the accuracy of formulas utilized for pediatric eyes.

Recently, several original studies have been published on the topic of IOL power calculation in children, with only a few review ones. Kaur et al. published a narrative review in 2021 [22]. They mainly discussed in detail the differences between IOL calculation in children and in adults. They also tried to find the most accurate IOL power calculation formula for children, however, they considered only the second- (SRK II), third- (Hoffer Q, Holladay 1, SRK/T), and fourth-generation formulas (Haigis, Barrett Universal II), ignoring the latest formulas. A systematic review including 12 articles was developed by Zhong et al. in 2021 [9]. However, only four of the cited papers had been published after 2019. Besides, the accuracy of essentially only five IOL power calculation formulas (SRK II, Holladay 1, Hoffer Q, SRK/T, Holladay 2) was considered because Haigis and Barrett Universal II were included in only two articles. The same formulas were included in the most recent Bayesian network meta-analysis by Hong et al. [23]. However, the paper is in Chinese.

Our study depicts in detail the 12 formulas most often used in IOL power calculation in children. The precise and clear methodology follows the Preferred Reporting Items for Systematic Reviews and Meta-Analysis (PRISMA) guidelines. The comparison of formula accuracy is based on carefully selected articles, as many as 13 of which were published after 2019. Finally, clear tables make visualizations much easier.

## 2. Methods

The methodology follows PRISMA guidelines. This review includes papers concerning the accuracy of IOL power calculation formulas in children eyes that were published from 2019–2024. The papers were identified by a literature search of medical and other databases (Pubmed/MEDLINE, Crossref, Google Scholar) using the combination of the following key words: “IOL power calculation formula”, “pediatric cataract”, “congenital cataract”, “pediatric intraocular lens implantation”, “lens power estimation”, “IOL power selection”, “phacoemulsification”, “Hoffer Q”, “SRK/T”, “Barrett Universal II”, “Hill-RBF”, and “Kane”. Duplicates were eliminated during the screening process. The initial search phase focused on abstracts. Only peer-reviewed articles in English presented as original studies or reviews and meta-analyses were considered, while editorials were excluded. After the preliminary search, only 14 papers were chosen for further analysis. The articles were not differentiated in terms of how biometric data were obtained. The studies with the maximum sample sizes (≥40 eyes), the greatest number of compared IOL power calculation formulas (≥3) and the most recent publication dates (from the last 5 years) were utilized. The risk of bias in the articles was assessed. For this purpose, the following were considered: random-sequence generation and allocation concealment (selection bias), blinding of participants and personnel (performance bias), blinding of outcome assessment (detection bias), incomplete outcome data (attrition bias), and selective reporting (reporting bias). Papers with high risk of bias were excluded. However, contrary to Hoffer’s protocol, articles were included in which both eyes of a given patient were examined [24].

### Ethics

This article is based on previously conducted studies and does not contain any new studies with human participants or animals performed by any of the authors.

## 3. Results

**Figure 1:**
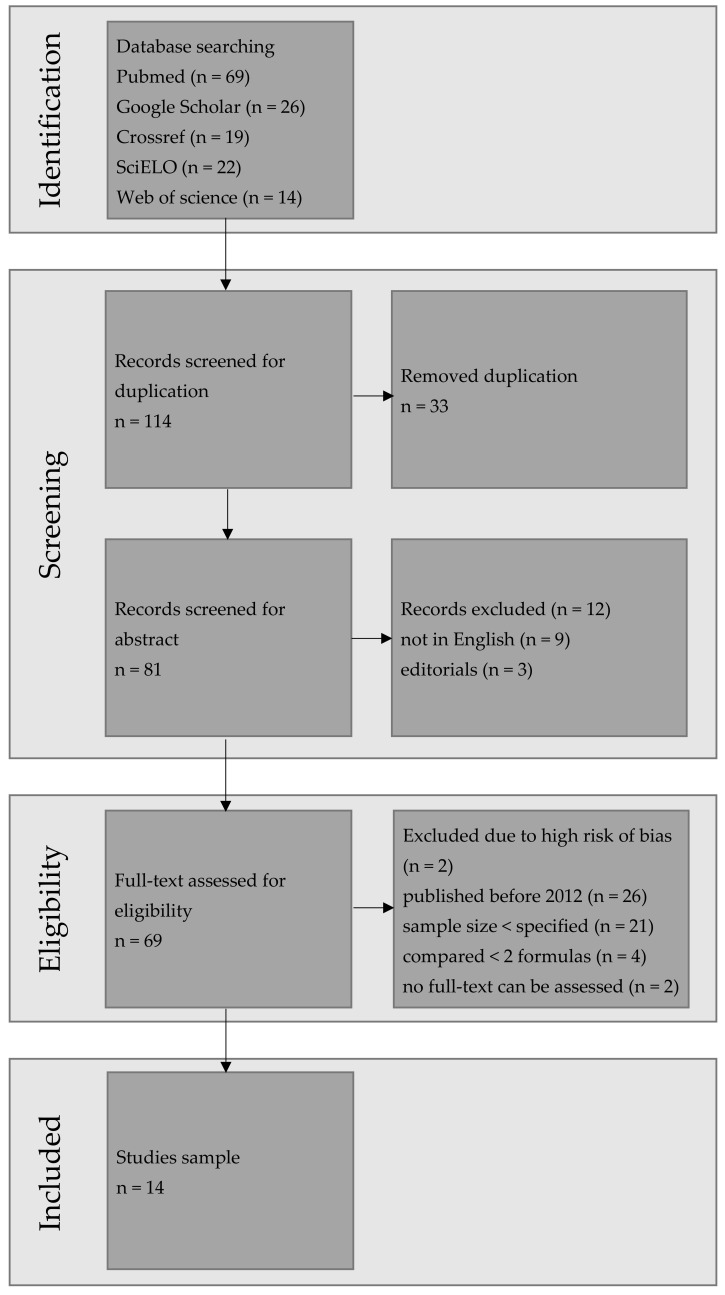
The PRISMA flow chart.

After duplicates had been eliminated, 81 articles were retrieved and subjected to analysis. Following an initial search, 14 papers were selected for further scrutiny. The detailed PRISMA flow chart (Figure 1) illustrates the process of identification, screening, and final selection of papers for this review. It was observed that various formulas exhibit specific advantages and disadvantages in terms of patient age. Several tools can be employed to evaluate the accuracy of the selected formula. Most studies typically assess mean absolute error (MAE) and the percentage of eyes within ±0.5 D, while median absolute error (MedAE) is less commonly used. Hoffer et al. recommended MedAE as a primary outcome due to the non-normal distribution of absolute refractive prediction errors [24]. Cooke et al. suggested a mean rank score for subgroup analysis, such as one based on axial length (AL) [25], while Haigis introduced his own ranking system [24]. More recently, studies have increasingly relied on root mean square absolute error (RMSAE) as an outcome [26,27,28]. RMSAE values are compared between formulas using the bootstrap-t method with Holm sequential correction [27].

### 3.1. Formulas Description

**1.**  **The Holladay 1 formula**

The Holladay 1 formula was launched in 1988 by Jack T. Holladay as the following mathematical formula:

IOL Power = A constant + [1.336 − (K/1000) − (0.333 ∗ AL)] − R

A constant—lens-specific constant
R—desired post-operative refraction

It was based on the variables AL, K and specific surgeon factor (SF). The formula yielded accurate outcomes for short eyes [29].

**2.**  **The SRK/T formula**

The SRK/T formula, named after its developers Donald R. Sanders, John A. Retzlaff, and Manus C. Kraff, with “T” for “theoretical”, was published in 1990. This formula integrates a linear regression approach with a theoretical eye model to enhance prediction accuracy. Through iterative refinement on five datasets encompassing 1677 cases with posterior chamber lenses, it optimizes predictions of postoperative ACD, retinal thickness, AL correction, and corneal refractive index. One of its notable advantages is its adaptability: it can be computed using empirically derived SRK A-constants accumulated over the years or by utilizing estimates of ACD. The variables incorporated into the SRK/T formula include AL, K, and ACD [30].

**3.**  **The Hoffer Q formula**

Kenneth J. Hoffer published the Hoffer Q formula in 1993. It was developed to predict the pseudophakic ACD for theoretic IOL power formulas. It relies on AL, K and personalized ACD [31]. It uses the following mathematical formula:

IOL Power = 1336/(AL − ACD – 0.05) − 1336/[(1336/K + r) − (ACD + 0.05/1000)]

r—corneal radius

This formula was recommended for short eyes [32,33].

**4.**  **The Holladay 2 formula**

The Holladay 2 formula, launched in 1996, determines ELP using seven parameters, i.e., AL, average K, horizontal white-to-white (WTW), preoperative refraction, ACD, lens thickness (LT) and age (in order of importance). This formula was the result of a comprehensive study involving 34 cataract surgeons from around the world. A large dataset of 34,000 eyes was collected and analyzed to determine the relative significance of each variable. The formula uses an ACD of 5.601 to set up a specific lens constant [34].

**5.**  **The Haigis formula**

In 2000, Wolfgang Haigis introduced the Haigis formula, an empirical calculation method that incorporates AL, K, ACD, and three constants: a0, a1, and a2. These constants serve to adjust the predicted power of the IOL based on observed outcomes specific to a surgeon and the individual anatomical characteristics of the eye [29]. Similar to the SRK/T A-constant, a0 shifts the power prediction curve, while a1 and a2 are associated with the measured ACD and AL, respectively. This approach allows the formula to produce tailored IOL power adjustments, enhancing precision in refractive outcomes.

**6.**  **The Olsen formula**

The Olsen formula, established by Thomas Olsen in 2006, leverages advanced ray-tracing techniques to accurately model the true physical dimensions of the eye’s optical system. This formula precisely forecasts the IOL position by employing the innovative C-constant concept, which represents the ratio according to which the empty capsular bag will encapsulate and fixate an IOL following in-the-bag implantation. The unique methodology of the Olsen formula assesses the IOL position based on preoperative ACD and LT, making traditional factors such as AL, K, WTW, IOL power, age, and gender unnecessary. A notable advantage of the Olsen formula is its ability to accurately determine the implant power for any eye type, including for eyes that have undergone refractive surgery, relying solely on precise measurements of ACD and LT. Furthermore, the Olsen formula integrates modern computational techniques and personalized eye models, enhancing its accuracy and reliability [35].

**7.**  **The T2 formula**

In 2010, Richard M. Sheard, Guy T. Smith, and David L. Cooke introduced the T2 formula, an adaptation of the SRK/T formula designed to enhance the original method, particularly in cases of extreme keratometry (K). The T2 formula was developed to address the non-physiological behavior observed in corneal-height prediction, known as the “cusp phenomenon.” It retains the same optical A-constant as the SRK/T formula and employs an intensified regression algorithm for postoperative prognosis of the anterior segment of the eye. The T2 formula was programmed into Excel based on the original data. The cusp phenomenon refers to an abnormality wherein the predicted corneal height does not follow a smooth physiological curve, leading to potential inaccuracies in IOL power calculations. By refining the SRK/T formula, the T2 formula aims to provide more accurate IOL power predictions, thus improving visual outcomes for patients, especially those with atypical corneal curvatures, undergoing cataract surgery [36].

**8.**  **The Barrett Universal II formula**

The Barrett Universal II formula, introduced in 2010 by Graham Barrett, is grounded in the Gaussian simplification of Snellen’s refraction law within the paraxial space. This formula examines the variations in principal planes associated with different IOL powers. It also accounts for the shift in optic configuration from a biconvex lens to a meniscus lens. Furthermore, it considers the changes in vergence that occur when the lens transitions from a positive to a negative value, thereby eliminating the need for additional correction factors or constants tailored for highly myopic patients with extended AL. The Barrett Universal II formula employs a distinct theoretical model to predict the ELP, a respect in which it significantly diverges from other existing models. As a theoretical framework, it utilizes traditional mathematical principles for IOL power calculation based on essential data such as the lens factor or A-constant, AL, K, ACD, and target refraction. Optional parameters like LT and WTW measurements are also considered. Notably, it has been demonstrated that the Barrett formula can perform calculations even in the absence of ACD measurements [29].

**9.**  **The Ladas Super Formula AI**

In 2015, John G. Ladas, Albert Jun, Aazim Siddiqui, and Uday Devgan introduced the concept of an IOL “super formula” that integrates the strengths of several existing models. They analyzed the Hoffer Q, Holladay 1, Holladay 1 with Koch adjustment, SRK/T, and Haigis formulas, treating each as a two-dimensional algebraic equation. Ladas et al. innovatively described these formulas as three-dimensional mathematical equations graphed along the AL (mm), corneal power (D), and IOL power (D) axes, enabling a comprehensive analysis of their accuracy. By synthesizing data from the peer-reviewed literature and identifying the optimal characteristics of each formula, they developed an IOL “super surface” [37]. Subsequent retrospective analyses indicated that SRK/T tends to perform better for longer eyes [38], whereas Hoffer Q demonstrates greater accuracy for very short eyes [32]. Additionally, eyes with specific AL or corneal powers often require further optimization; for instance, hyperopic eyes pose challenges due to the significant impact of minor changes in ELP on lens power calculations. From this “super surface”, the Ladas formula was derived, representing an advanced approach to IOL power calculation. The final step in their methodology involved refining existing formulas through deep learning techniques, aiming to enhance precision and adaptability in clinical applications [39].

**10.**  **The Hill−Radial Basis Function (RBF) formula**

The Hill−RBF formula, introduced in 2016 by Warren E. Hill, MD, represents the first AI-based IOL calculation model and utilizes radial basis functions. This innovative approach is entirely data-driven, leveraging pattern-recognition techniques developed in MATLAB and advanced data-interpolation methodologies. Version 2.0 of the Hill-RBF formula utilized a substantial dataset of more than 12,000 eyes, whereas version 3.0, available since September 2020, expanded this dataset to include over 30,000 eyes. Initially, Hill−RBF would abstain from providing a refractive prediction if accuracy was uncertain, particularly in early versions limited to plano target refraction. Presently, the formula always delivers a prediction, which is accompanied by a cautionary indicator in cases in which the outcome may be uncertain. Key inputs for the Hill−RBF 3.0 formula include AL, K, ACD, LT, WTW, CCT, and gender [40]. This comprehensive dataset and algorithmic approach mark significant advancements in personalized IOL power calculation.

**11.**  **The Kane formula**

Jack X Kane, MD, an Australian, introduced a novel IOL power calculation formula in September 2017, drawing from a dataset of approximately 30,000 meticulously curated cases sourced from selected surgeons. The Kane formula integrates theoretical optics, thin-lens formulas, and “big data” methodologies to enhance its predictive accuracy. As a hybrid model, it incorporates elements of regression analysis and AI to further refine outcomes. Development of the Kane formula utilized high-performance cloud-based computing, leveraging the ability of the cloud infrastructure to function as a virtual supercomputer to accelerate data processing. A primary objective of developing this formula was to mitigate errors observed in cases with extreme AL. The formula necessitates the inclusion of the A-constant (similar to the SRK/T A-constant, though optimized by the surgeon), AL, K, ACD, and patient gender as mandatory variables. Additionally, LT and CCT can be optionally inputted to enhance predictive accuracy. This inclusive approach allows users of older biometric devices to adopt the Kane formula effectively [41]. The combination of advanced computational techniques and a robust dataset underscores the Kane formula’s contribution to personalized IOL power calculation.

**12.**  **The Emmetropia Verifying Optical (EVO) formula**

EVO, developed in 2019 by Tun Kuan Yeo of Singapore, introduces a novel thick-lens formula rooted in the concept of emmetropization theory. This approach seeks to optimize postoperative refractive outcomes by considering individual eye characteristics through an “emmetropia factor.” Unlike traditional formulas, EVO integrates sophisticated algorithms designed to accommodate a range of IOL geometries and powers, aiming to enhance accuracy across diverse patient profiles. While specific details regarding the inclusion of AI components in EVO have not been disclosed publicly, Version 2.0 of the formula is accessible through the dedicated website www.evoiolcalculator.com (accessed on 26 June 2024). This version emphasizes AL, K, and ACD as fundamental predictors for calculating IOL power. Additionally, optional parameters such as LT and CCT can be inputted to refine predictions further. Recent studies have validated EVO’s robustness by demonstrating its ability to generate accurate predictions even when ACD measurements are unavailable. Version 2.0 has been specifically noted for its improved accuracy in cases involving extreme AL (short and long eyes) and in the calculation of toric IOLs, highlighting its versatility and reliability in clinical practice [29]. The EVO formula calculator serves as a valuable tool for ophthalmologists and eye-care professionals seeking precise and personalized IOL power calculations based on contemporary theoretical foundations and empirical data.

### 3.2. Accuracy of IOL Calculation Formulas in Children

**Figure 2:**
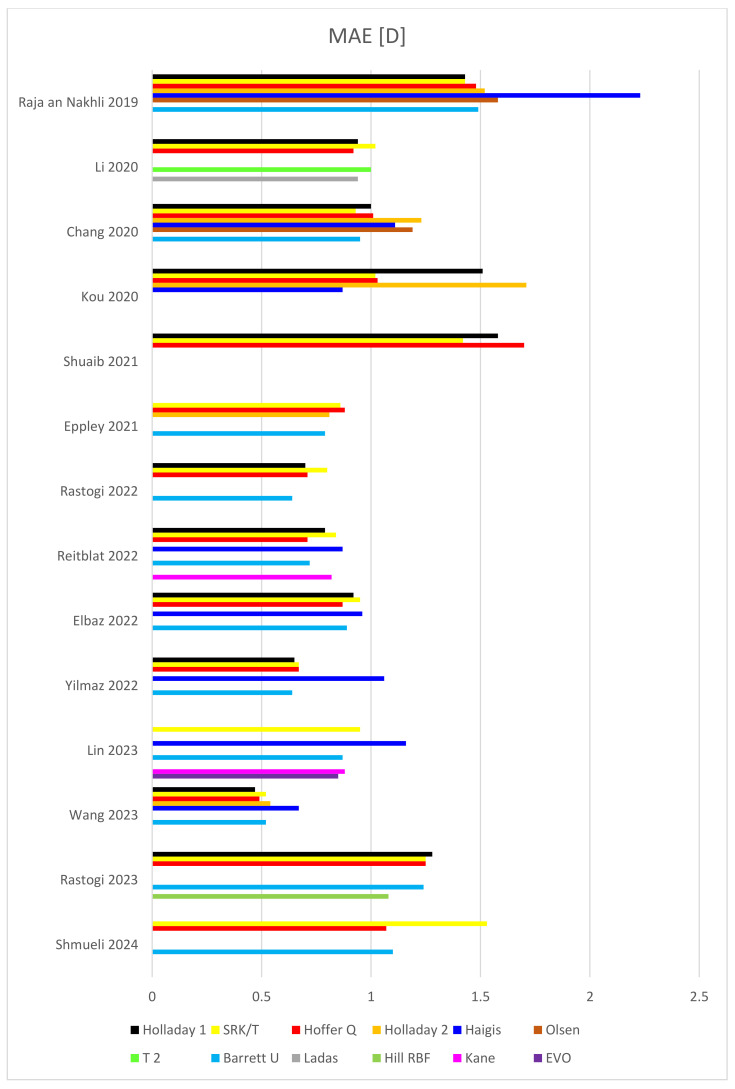
Outcomes of MAE in children’s eyes in several recent studies [7,8,17,18,19,20,21,42,43,44,45,46,47,48].

**Figure 3:**
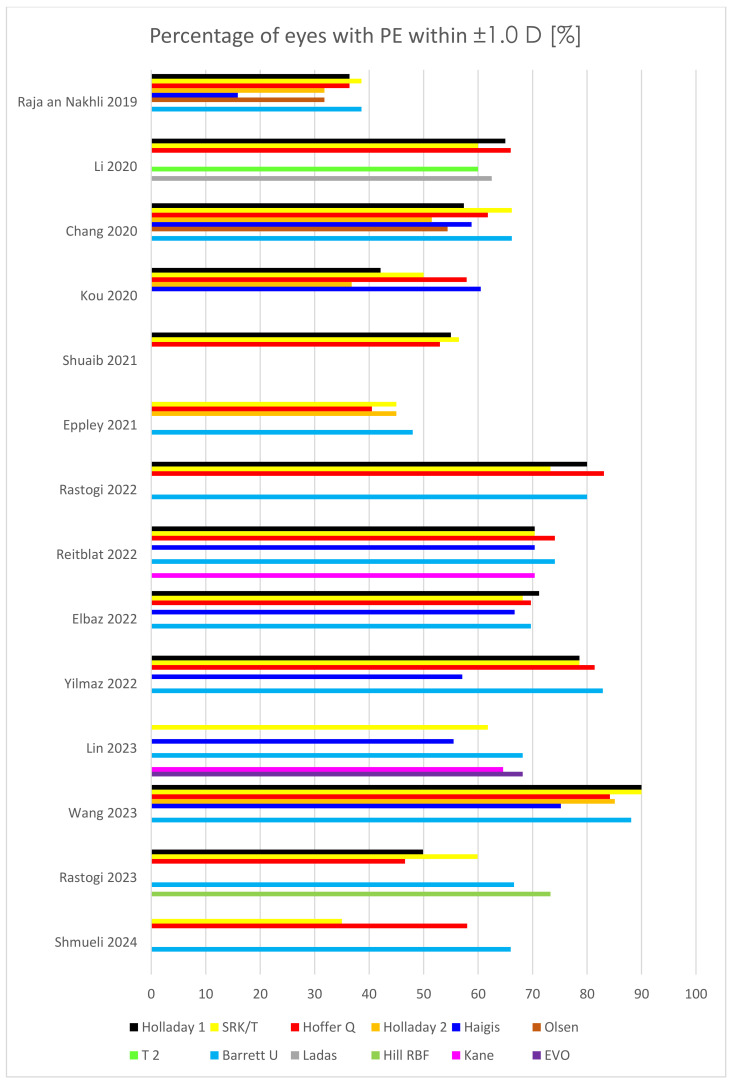
Outcomes of percentage of eyes with prediction error (PE) within ±1.0 D in children’s eyes in several recent studies [7,8,17,18,19,20,21,42,43,44,45,46,47,48].

The comprehensive findings from multiple peer-reviewed studies investigating the utilization of intraocular lens (IOL) power calculation formulas specifically in pediatric eyes are consolidated and presented in Table 1, Figure 2 and Figure 3. Demographic characteristics of the patients are listed in Table 2.

The bias risk was evaluated for each study, as shown in Table 3.

## 4. Discussion

Currently, the main challenge in pediatric cataract surgery is not the surgical technique or the IOL used, but the postoperative refractive error. Amblyopia, which can occur due to post-operative refractive error, destroys the benefits gained even from near-perfect and timely surgery. Even if we settle on what the ideal postoperative target refraction should be in a child, we can still be surprised by postoperative refractive errors. So, choosing the right IOL power calculation formula is fundamental.

In 2020 Li et al. published study based on 377 eyes of 377 pediatric patients. They compared the accuracy of six formulas, including newer formulas, i.e., Ladas Super and T2 [18]. In terms of MAE, Hoffer Q achieved the best result, followed by Ladas Super and Holladay 1 (0.92, 0,94, 0,94; respectively). In addition, Hoffer Q yielded the highest percentage of patients with PE within ±1.0 D, placing it ahead of Holladay 1 and Ladas Super (66, 65, and 62.5, respectively). The large sample size and comparison of pediatric patients with various AL values and ages are advantages of this study. Its limitations are ambiguous. Multiple IOL brands and types were used, and various surgeons performed phacoemulsification. However, according to some authors, such a design makes the results generalizable [34]. Undoubtedly, its retrospective design and use of applanation biometry are limitations of this study.

Shuaib et al., in their 2021 study, included 308 eyes of 255 children and compared four IOL power calculation formulas [44]. SRK/T, placing it ahead of Holladay 1 and Hoffer Q, yielded the smallest MAE (1.42, 1.58 and 1.70, respectively). These three formulas also yielded the best outcomes in terms of percentage of patients with PE within ±1.0 D (56.5, 55.0, 53.0, respectively). However, the method of obtaining the biometric data (contact A-scan biometry) is the greatest limitation of the study. Other limitations were the use of only four formulas (second- and third-generation), the retrospective design and the use of a procedure that did not follow the Hoffer recommendations (53 patients had both eyes included in the study) [49]. The participation of many surgeons in the study may have resulted in more generalized outcomes.

Recently, Shmueli et al. published a study involving 151 pediatric eyes of 104 patients. Hoffer Q, followed by Barrett Universal II and SRK/T, achieved the best results in terms of MAE (1.07, 1.10, 1.53, respectively). In turn, Barrett Universal II yielded the highest percentage of patients with PE within ±1.0 D, placing it ahead of Hoffer Q and SRK/T (66.0, 58.0, 35.0, respectively). However, this study has several limitations. First, bilateral cases were included (47 patients). Second, the authors compared the accuracy of only three IOL power calculation formulas. Third, the A constant of IOL used in this study was derived from adults due to the lack of an optimized A constant for children. Fourth, most axial length measurements were done by contact rather than by immersion sonography, which affects accuracy. Fifth, due to the retrospective nature of this study, the groups were not balanced concerning AL [8].

Our study is a review based on 14 articles and 1682 eyes. We have considered the accuracy of 12 IOL power calculation formulas, excluding SRK II, which is sometimes still used; however, as a second-generation formula, it is less precise [23].

In terms of MAE, in 14 studies, Hoffer Q yielded the best outcome four times (total 693 eyes) [8,17,18,21], while SRK/T performed best three times (total 420 eyes) [19,42,44], as did Barrett Universal II (194 eyes). Barrett Universal II yielded the second-best result as many as six times (total 556 eyes) [8,17,19,20,21,48], but Holladay 1 did so in only four studies, although these studies included as many as 815 eyes) [18,44,46,47]. On the other hand, Haigis obtained the worst MAE (total 453 eyes) in 6 studies [7,17,20,42,47,48], and SRK/T did so in four, which, however, included as many as 698 eyes [8,18,46,48].

Considering patients with PE within ±1.0 D, Barrett Universal II yielded the highest percentage as many as seven times (total 569 eyes) [8,19,20,42,45,47,48], and SRK/T did so four times (total 521 eyes) [7,19,42,44]. Hoffer Q achieved the second-highest percentage in six studies (total 501 eyes) [8,17,19,42,43,47], and Holladay 1 in 5 which, however, included as many as 851 eyes [18,20,42,44,46]. Although Haigis yielded the lowest percentage as many as 5 times which only represented 391 eyes [7,17,42,47,48], but SRK/T 3 times, however, total as many as 588 eyes [8,18,46], and Hoffer Q also 3 times (total 471 eyes) [21,44,45].

Thus, the study showed the highest accuracy of the Barrett Universal II formula. Very robust outcomes are given by Holladay 1. Hoffer Q and SRK/T obtain accurate results, however, give postoperative refractive surprises often. Hill-RBF and EVO may provide accurate outcomes, however, studies using these formulas are rare, so further examinations are needed.

Twelve observational studies, involving 1647 pediatric eyes (1102 eyes were calculated using Holladay 1, 439 with Holladay 2, 1647 with Hoffer Q, 1647 with SRK/T and 1319 with SRK II) were enrolled in Zhong et al. 2021 meta-analysis. The best MAE was yielded by Holladay 1, followed by Holladay 2 and Hoffer Q (0.97, 1.05, 1.05; respectively). SRK II yielded the worst MAE (1.34). Good outcomes from the Holladay 1 formula are in line with the results of our study because they did not consider Barrett Universal II. However, this meta-analysis has several limitations. First, they compared only five IOL power calculation formulas, excluding Haigis, Barrett Universal II, and Kane. Second, there was moderate-to-substantial heterogeneity observed in some analyses. Third, the pooled results were mainly based on retrospective cohort studies, which are subject to inevitable selection bias and confounding. Fourth, due to the significant variability in the study sample (age, AL), they failed to reach a definitive conclusion for the whole study sample [9].

The narrative review published by Kaur et al. in 2021 focuses on challenges in and approaches to IOL power calculation in children and does not compare the accuracy of IOL power calculation formulas. They wonder which formula is the least inaccurate, rather than which formula is the most accurate. They point out that accuracy of the advanced theoretical formulas is low for pediatric cataract surgery because the current IOL power calculation formulas largely originated from and were optimized based on studies in adults [22].

A recently published (2023) Bayesian network meta-analysis by Hong et al. comprised 13 studies of 1781 eyes and eight formulas. The study proved that the Barrett Universal II, Holladay 1, and SRK/T formulas provide more accuracy for IOL power calculation in pediatric cataract eyes. It is difficult to discuss in detail the methods, results, and limitations of this study because the article is in Chinese [23].

Our study has some limitations. First, we based this study on MAE and the percentage of patients within ±0.50 and ±1.00; however, many authors prefer MedAE [24]. Second, the method of obtaining the biometric data was not considered. Third, preliminary sample-size calculation was not included. Finally, no meta-analysis was performed. However, the authors concluded that heterogeneity of included studies was too great (various equipment utilized to achieve biometric data, different IOLs, varied devices used for obtaining postoperative refraction, etc.) and that considering all these factors would significantly limit the statistical analysis.

## 5. Conclusions

Accurately calculating IOL power in children remains a challenge. This is due to the different structure of the pediatric eye, the difficulties in obtaining biometric data, the variable target refraction that depends on the child’s age, and most importantly, the lack of child-specific IOL power calculation formulas.

Our study shows that the Barrett Universal II formula most often yielded the best refractive outcomes in pediatric eyes [8,17,19,20,21,48]. The formula is readily available because it is on the equipment of the latest biometric measuring devices e.g., Zeiss IOLMaster 700 (carl Zeiss Meditec AG, Jena, Germany). In addition, an online Barrett’s calculator is accessible on the European Society of Cataract & Refractive Surgeons website. Very good postoperative results can also be obtained using the Holladay 1 formula [18,44,46,47]. Holladay 1 is available on most biometric appliances. In clinical practice, the Holladay 1 formula can guide clinicians in achieving relatively accurate calculation outcomes when the hardware environment is limited and has a certain value in clinical application. Newer formulas, e.g., Hill−RBF or EVO, are relatively rarely used in children, but their results are promising.

Although there is still no consensus among cataract surgeons on which formula to choose for calculating IOL power and each pediatric patient should be treated individually, our study shows that with both the latest biometric measuring devices and slightly older equipment, fairly accurate results can be obtained in IOL power calculation for children.

## Figures and Tables

**Table 1:** Outcomes of MAE and percentage of eyes with PE within ±0.5 D and ±1.0 D in children’s eyes in several recent studies.

Study		Holl 1	SRK/T	Hoff Q	Holl 2	Haigis	Olsen	T 2	Barr Uni	Ladas	Hill RBF	Kane	EVO
Raja an Nakhli 2019 (44 eyes) [42]	MAE	1.43	1.43	1.48	1.52	2.23	1.58		1.49				
	±0.5 D [%]	25.0	15.9	22.7	15.9	6.8	11.4		13.6				
	±1.0 D [%]	36.4	38.6	36.4	31.8	15.9	31.8		38.6				
Li 2020 (377 eyes) [18]	MAE	0.94	1.02	0.92				1.0		0.94			
	±0.5 D [%]	37.0	36.0	38.0				37.5		36.5			
	±1.0 D [%]	65.0	60.0	66.0				60.0		62.5			
Chang 2020 (68 eyes) [19]	MAE	1.00	0.93	1.01	1.23	1.11	1.19		0.95				
	±0.5 D [%]	28.0	38.2	29.4	23.6	38.2	26.5		36.8				
	±1.0 D [%]	57.4	66.2	61.8	51.5	58.8	54.4		66.2				
Kou 2020 (102 eyes) [43]	MAE	1.51	1.02	1.03	1.71	0.87							
	±0.5 D [%]	18.4	26.3	23.7	18.4	34.2							
	±1.0 D [%]	42.1	50.0	57.9	36.8	60.5							
Shuaib 2021 (308 eyes) [44]	MAE	1.58	1.42	1.70									
	±0.5 D [%]	35.0	37.0	32.5									
	±1.0 D [%]	55.0	56.5	53.0									
Eppley 2021 (64 eyes) [45]	MAE		0.86	0.88	0.81				0.79				
	±0.5 D [%]		32.0	34.0	34.0				36.0				
	±1.0 D [%]		45.0	40.5	45.0				48.0				
Rastogi 2022 (60 eyes) [46]	MAE	0.70	0.80	0.71					0.64				
	±0.5 D [%]	50.0	46.7	46.7					56.7				
	±1.0 D [%]	80.0	73.3	83.1					80.0				
Reitblat 2022 (62 eyes) [20]	MAE	0.79	0.84	0.71		0.87			0.72			0.82	
	±0.5 D [%]	44.4	29.6	51.9		51.9			55.6			44.4	
	±1.0 D [%]	70.4	70.4	74.1		70.4			74.1			70.4	
Elbaz 2022 (66 eyes) [17]	MAE	0.92	0.95	0.87		0.96			0.89				
	±0.5 D [%]	42.4	31.8	51.5		47.0			51.5				
	±1.0 D [%]	71.2	68.2	69.7		66.7			69.7				
Yilmaz 2022 (70 eyes) [47]	MAE	0.65	0.67	0.67		1.06			0.64				
	±0.5 D [%]	52.9	47.1	52.9		30.0			61.4				
	±1.0 D [%]	78.6	78.6	81.4		57.1			82.9				
Lin 2023 (110 eyes) [48]	MAE		0.95			1.16			0.87			0.88	0.85
	±0.5 D [%]												
	±1.0 D [%]		61.8			55.5			68.2			64.6	68.2
Wang 2023 (101 eyes) [7]	MAE	0.47	0.52	0.49	0.54	0.67			0.52				
	±0.5 D [%]	62.4	58.4	62.4	62.4	50.5			59.4				
	±1.0 D [%]	90.0	90.0	84.2	85.1	75.2			88.1				
Rastogi 2023 (99 eyes) [21]	MAE	1.28	1.25	1.25					1.24		1.08		
	±0.5 D [%]	16.6	13.3	23.3					30.0		43.3		
	±1.0 D [%]	49.9	59.9	46.6					66.6		73.3		
Shmueli 2024 (151 eyes) [8]	MAE		1.53	1.07					1.10				
	±0.5 D [%]		10.0	35.0					34.0				
	±1.0 D [%]		35.0	58.0					66.0				

MAE: mean absolute error.

**Table 2:** Demographic characteristics of the patients.

Study		Age (y)	IOL Power (D)	AL (mm)	Km (D)	ACD (mm)	LT (mm)	CCT (μm)	WTW (mm)
Raja an Nakhli [42]	Mean		21.16	21.87	44.47	3.57	3.73		
	SD		3.79	1.47	2.67	1.47	0.89		
	Median	2.85	21.50	21.68	44.16	3.64	3.67		
	Min	2.04	13.50	17.99	40.50	2.22	2.58		
	Max	6.14	30.00	24.13	53.50	4.45	5.45		
Li [18]	Mean	4.60	22.56	22.48	43.95				
	SD	2.33	5.04	1.91	2.01				
	Median								
	Min	0.75	4.0	17.85	37.75				
	Max	12.5	34.0	31.46	53.63				
Chang [19]	Mean	1.94	25.36	20.15	43.88	2.99	3.78	543.2	10.47
	SD	1.35	2.74	0.74	2.72	0.49	0.48	50.25	0.67
	Median								
	Min								
	Max								
Kou [43]	Mean	3.36	22.87	21.96	43.96	3.05			10.85
	SD								
	Median								
	Min	2.0	12.0	19.44	41.23	2.26			9.80
	Max	4.5	28.0	25.85	49.81	3.73			12.40
Shuaib [44]	Mean	4.74		22.01	43.42				
	SD	3.19		1.93	3.57				
	Median								
	Min	0.8		17.45	37.25				
	Max	14.0		30.00	57.56				
Eppley [45]	Mean	5.90	20.86	22.55	44.43	3.43	4.08	555.65	11.68
	SD	3.56	6.13	1.62	2.07	0.51	0.89	49.77	0.77
	Median	5.37	21.00	22.23	44.35	3.41	3.72	557.50	11.78
	Min	1.46	7.00	19.64	39.39	2.27	3.00	468.00	10.00
	Max	15.45	33.00	26.30	48.75	4.40	6.49	672.00	14.00
Rastogi [46]	Mean	8.53		22.75	44.34				
	SD	3.43		1.39	1.82				
	Median								
	Min	5		20.64	39.12				
	Max	16		25.69	47.62				
Reitblat [20]	Mean	6.2	23.2	22.43	43.83	3.58			
	SD		5.1	1.66	2.06	0.44			
	Median	5.98	39.0				3.41		12.0
	Min	0.9	12.0	19.45	40.09	2.35	1.71		10.5
	Max	17.5		27.81	49.12	4.48	6.52		13.5
Elbaz [17]	Mean		23.3	22.3	43.9	3.6			
	SD		5.1	1.6	2.3	0.4			
	Median	6.2					3.4		12.0
	Min	0.9	12.0	19.5	40.5	2.4	1.7		10.5
	Max	17.5	39.0	27.8	50.3	4.5	6.5		13.1
Yilmaz [47]	Mean	7.9	25.8	22.27	43.74	3.76		571.5	
	SD	3.6	3.5	1.19	1.98	0.41		33.6	
	Median								
	Min	3.0	18	19.78	39.25	2.6		500	
	Max	15.0	34	25.94	49.54	4.74		644	
Lin [48]	Mean	3.1							
	SD	1.9							
	Median								
	Min								
	Max								
Wang [7]	Mean	5.99	23.35	22.58	44.01	3.26			
	SD	3.15	5.02	1.48	2.12	0.43			
	Median								
	Min	3	9.0	19.79	40.40	1.88			
	Max	14	33.5	28.26	50.31	3.97			
Rastogi [21]	Mean	6.5							
	SD								
	Median								
	Min	4							
	Max	18							
Shmueli [8]	Mean	6.27		21.8	44.1				
	SD	4.03		1.2	2.0				
	Median	6.17		21.6	44.7				
	Min	0.83		19.5	39.7				
	Max	16.25		25.9	48.3				

**Table 3:** The risk of bias of included studies.

Study	Bias				
	Selection	Performance	Detection	Attrition	Reporting
Raja an Nakhli [42]	++	―	―	―	??
Li [18]	―	―	―	―	―
Chang [19]	―	―	―	―	??
Kou [43]	++	―	―	―	++
Shuaib [44]	―	―	++	―	―
Eppley [45]	―	―	??	―	―
Rastogi [46]	++	―	―	―	―
Reitblat [20]	―	―	??	―	―
Elbaz [17]	―	―	++	―	??
Yilmaz [47]	―	++	??	―	―
Lin [48]	++	―	―	??	―
Wang [7]	++	―	―	―	++
Rastogi [21]	++	??	―	―	―
Shmueli [8]	++	―	―	―	??

## Data Availability

No new data were created or analyzed in this study. Data sharing is not applicable to this article.
